# Effects of Camphorquinone on Cytotoxicity, Cell Cycle Regulation and Prostaglandin E_2_ Production of Dental Pulp Cells: Role of ROS, ATM/Chk2, MEK/ERK and Hemeoxygenase-1

**DOI:** 10.1371/journal.pone.0143663

**Published:** 2015-12-14

**Authors:** Mei-Chi Chang, Li-Deh Lin, Min-Tsz Wu, Chiu-Po Chan, Hsiao-Hua Chang, Ming-Shu Lee, Tzu-Ying Sun, Po-Yuan Jeng, Sin-Yuet Yeung, Hsueh-Jen Lin, Jiiang-Huei Jeng

**Affiliations:** 1 Biomedical Science Team, Chang Gung University of Science and Technology, Kwei-Shan, Taoyuan City, Taiwan; 2 Laboratory of Dental Pharmacology, Toxicology & Material Biocompatibility, Graduate Institute of Clinical Dentistry, and National Taiwan University Medical College, Taipei, Taiwan; 3 Department of Dentistry, National Taiwan University Hospital, Taipei, Taiwan; 4 Department of Dentistry, Chang Gung Memorial Hospital, Taipei, Taiwan; 5 School of Dentistry, University of Cardenal Herrera, CEU, Valencia, Spain; 6 Department of Dentistry, Show Chwan Memorial Hospital, Chang-Hua, Taiwan; National Cheng Kung University, TAIWAN

## Abstract

Camphorquinone (CQ) is a popularly-used photosensitizer in composite resin restoration. In this study, the effects of CQ on cytotoxicity and inflammation-related genes and proteins expression of pulp cells were investigated. The role of reactive oxygen species (ROS), ATM/Chk2/p53 and hemeoxygenase-1 (HO-1) and MEK/ERK signaling was also evaluated. We found that ROS and free radicals may play important role in CQ toxicity. CQ (1 and 2 mM) decreased the viability of pulp cells to about 70% and 50% of control, respectively. CQ also induced G_2_/M cell cycle arrest and apoptosis of pulp cells. The expression of type I collagen, cdc2, cyclin B, and cdc25C was inhibited, while p21, HO-1 and cyclooxygenase-2 (COX-2) were stimulated by CQ. CQ also activated ATM, Chk2, and p53 phosphorylation and GADD45α expression. Besides, exposure to CQ increased cellular ROS level and 8-isoprostane production. CQ also stimulated COX-2 expression and PGE_2_ production of pulp cells. The reduction of cell viability caused by CQ can be attenuated by N-acetyl-L-cysteine (NAC), catalase and superoxide dismutase (SOD), but can be promoted by Zinc protoporphyin (ZnPP). CQ stimulated ERK1/2 phosphorylation, and U0126 prevented the CQ-induced COX-2 expression and prostaglandin E_2_ (PGE_2_) production. These results indicate that CQ may cause cytotoxicity, cell cycle arrest, apoptosis, and PGE_2_ production of pulp cells. These events could be due to stimulation of ROS and 8-isoprostane production, ATM/Chk2/p53 signaling, HO-1, COX-2 and p21 expression, as well as the inhibition of cdc2, cdc25C and cyclin B1. These results are important for understanding the role of ROS in pathogenesis of pulp necrosis and pulpal inflammation after clinical composite resin filling.

## Introduction

In dentistry, resin composites are widely used as restorative materials because of their ease of handling and esthetic enhancement. The commonly used oligomers and monomers in organic polymer matrix of resin composites belong to dimethacrylates, which contain reactive carbon double bonds. They undergo free-radical polymerization that is a kind of addition polymerization, and polymerization initiators are contained to produce free radicals for initiating the reaction. The polymerization initiators used for light-cured resin composites usually consist of a photosensitizer, primarily camphorquinone (CQ), and a reducing agent which is often a tertiary amine such as dimethylaminoethyl methacrylate (DMAEMA) or dimethyl-para-toluidine (DMPT) [1].

The concentration of CQ in the resin phase usually ranges from 0.17% to 1.03% w/w [2]. CQ has two carbonyl groups with non-bonding electrons, and the absorption spectrum of it is relatively broad between 400 and 550 nm in the blue region of visible light, with the maximum at 468 nm. CQ produces a pair of free radicals through proton abstraction [3]. The monomer-polymer conversion rate of resin composites varies approximately from 35% to 77% [4]. The residual monomers and additives are free to diffuse out from the cured materials. They may be released into surrounding tissues, and may have potential toxic effects. CQ was identified as one of the main released components in extracts of resin-based materials [4,5].

Initiating radicals may indiscriminately react with molecular oxygen forming reactive oxygen species (ROS), which may potentially cause oxidative damage to the cells’ macromolecules. Generally, CQ reveals a moderate cytotoxic effect compared to other photoinitiators and most resin (co)monomers [6]. Studies on CQ are limited comparing to those on resin (co)monomers. Masuki *et al*. reported a statistically significant finding of growth inhibition and G_0_/G_1_ cell cycle arrest in humn gingival fibroblasts (HGF) treated with 1 and 5 mM CQ for 24 hours. They also noted that exposure to 5 mM CQ increased the numbers of apoptotic/necrotic cells [1]. Engelmann *et al*. found that at concentrations higher than 1 mM, CQ caused a significant concentration-dependent increase of intracellular ROS in human pulp fibroblasts (HPF) within 90 minutes of exposure. Moreover, the ROS increase was associated with a moderate decrease of glutathione (GSH), the most important intracellular ROS-scavenger, after treatment by 5 mM CQ for 90 minutes [7]. Volk *et al*. treated HGF with CQ or CQ in combination with 0.5 mM N-acetylcysteine (NAC), a ROS-scavenger, for 3 hours. The data showed that at concentrations higher than 1.25 mM, CQ caused a significant concentration-dependent increase of intracellular ROS, which was only associated with a moderate glutathione (GSH) decrease at the highest concentration of 2.5 mM CQ. They also found that NAC reduced CQ-induced ROS formation [8]. However, influences of CQ on cell cycle and cell death in human dental pulp cells are not available in the literature. In addition, the changes of the related genes and proteins expression are still not clear nowadays. Hemeoxygenase (HO) is the rate-limiting enzyme of microsomal heme degradation pathway, and biliverdin, one of the final products, is further converted to bilirubin. HO has been suggested to function as a defense system against oxidative stress, since biliverdin or bilirubin produced locally in the body may act as physiological antioxidants. HO-1 is an inducible isoform in response to stress such as oxidative stress, hypoxia, heavy metals, cytokines, and so forth [9]. However, the role of HO-1 in regulation of CQ toxicity is not clear.

The cell cycle was divided into four distinct phases: G_1_, S, G_2_, and M. The transition from one cell cycle phase to another depends on a series of sequential events. The key regulatory proteins are the cyclin-dependent kinases (CDK) and their activating proteins, the cyclins. Different cyclin/CDK complexes are assembled and activated at different points of the cell cycle. CDK activity can be counteracted by cell cycle inhibitory proteins such as various CDK inhibitors (CKI). There are two different classes of CKI. The INK4 family includes p15, p16, p18, and p19, which specifically target CDK4 and CDK6. As for the Cip/Kip family, its family members consist of p21, p27, and p57. They inhibit a wide spectrum of CDKs [10]. Since cells are constantly under the attack of endogenous and exogenous damage, cells have evolved general mechanisms called checkpoints that monitor and confirm the successful completion of cell cycle events [11]. DNA damage may activate the checkpoint transducing kinases such as Ataxia telangiectasia mutated (ATM)/ ataxia-telangiectasia and Rad3 related (ATR) and checkpoint kinase 1 and 2 (Chk1/Chk2). Then, at the beginning of DNA damage, the acute and transient cell cycle delay is activated. Its downstream targets include cdc25A for G_1_/S, Nbs1 as well as SMC1 for intra-S, and cdc25C for G_2_/M phase. On the other hand, the p53-dependent delay and sustain of cell cycle arrest is slow-operating. Its targets are p21 for G_1_/S, and p21, 14-3-3σ as well as growth arrest and DNA damage inducible protein—GADD45 for G_2_/M phase [12]. Generally there are two types of cell death, namely, apoptosis and necrosis. When cytotoxic stimuli are intense, cells may get away from the cell cycle and undergo a programmed cell death called apoptosis. By contrast, necrosis is a kind of “cell murder” that follows the exposure of cells to a gross injury [1].

Clinically, some of the teeth may develop pulpitis and pulp necrosis after composite resin restoration. This is partly due to the toxicity of resin monomers or photo-initiator such as CQ. The purpose of this study is to investigate the influences of CQ with different concentrations on cytotoxicity to human dental pulp cells, including morphological changes, cell proliferation, cell cycle progression, cell death pattern and prostanoids production. Then, the expressions of cyclooxygenase-2 (COX-2), cell cycle regulation and apoptosis related genes and proteins under the treatment of CQ are evaluated. Besides, the roles of ROS, ATM/Chk2, HO-1 and MEK/extracellular signal-regulated kinase (ERK) in the CQ-induced cell changes are also evaluated.

## Materials and Methods

### Materials

CQ, dimethylsulfoxide (DMSO), 3-(4,5-dimethyl-thiazol-2-yl)-2,5-diphenyl-tetrazolium bromide (MTT), 2’,7’-dichlorodihydrofluorescein diacetate (DCFH-DA), propidium iodide (PI), N-acetylcysteine (NAC), catalase, superoxide dismutase (SOD) and Zinc protoporphyrin (ZnPP) were obtained from Sigma (Sigma Chemical Company, St Louis, MO, USA). The cell culture biological such as Dulbecco’s modified Eagle’s medium (DMEM), fetal bovine serum (FBS), penicillin and streptomycin were bought from Gibco (Life Technologies, Grand Island, NY, USA). Annexin V and reagents for flow cytometry were obtained from Becton Dickinson (Worldwide Inc., San-Jose, CA). RNA isolation kit and NucleoSpin RNA II were puchased from Macherey-Nagel (Macherey-Nagel Inc, Easton, PA, USA). The SuperScript^TM^ III First-Strain DNA Synthesis System for reverse transcriptase-polymerase chain reaction (RT-PCR) was from Invitrogen (Invitrogen Corporation, Carlsbad, CA, USA). The luminol reagents for western blotting and the primary antibodies Type I collagen, p-ERK, GAPDH, COX-2, HO-1, cdc2, cyclin B1, cdc25C, p-ATM, p-Chk2, p-p53, GADD45α were obtained from Santa Cruz Biotechnology (Santa Cruz Biotechnology Inc., Santa Cruz, CA, USA), and p21 was from GeneTex (Irvine, California, USA). Enzyme-linked immunosorbant assay (ELISA) kits for PGE_2_ and 8-isoprostane were from Cayman Chemical Company (Ann Arbor, MI, USA).

### Culture of Human Dental Pulp Cells

By the approval of Ethics Committee, National Taiwan University Hospital, three strains of human dental pulp cells were cultured from extracted premolars of patients under 25 years old with written informed consents by the patients or next of kin. Briefly, human dental pulp tissues were taken from caries- and periodontitis-free premolars extracted from three young donors (12–20 years old) for orthodontic purposes with proper written informed consent by the patients or next of kin on behalf of all minors enrolled in this study. Pulp cells were cultured by using a tissue explant technique [13–17]. Briefly a hammer was used to split the teeth in order to get the vital pulp tissues. Then a surgical knife was used to cut the dental pulp tissues into small pieces (about 1 mm^3^). The tissues were placed into 10-cm dishes and cultured by DMEM containing 10% FBS, 1x penicillin/streptomycin. When the growth of cells were about to reach the confluence, cells were detached from culture dishes by treatment with trypsin/ethylenediamine tetraacetic acid (EDTA) for subculture. The 3^rd^ to 8^th^ generations of the pulp cells were used in this study.

### Cytotoxicity of CQ on Human Dental Pulp Cells

In brief, 2.5 x 10^5^ human dental pulp cells were placed into 6-well culture plates with 2 ml of DMEM containing 10% FBS. After 24 hours of incubation, the culture medium was renewed by a fresh one containing 0.4% (v/v) DMSO (solvent) or various concentrations of CQ (0.1, 0.25, 0.5, 1, and 2 mM). After 24 hours of exposure, the morphological changes of human dental pulp cells were observed and photographed under a phase contrast microscope (Olympus IX 71, Olympus America Inc.). The culture medium was collected for analysis. Then the culture medium with MTT (0.5 mg/ml) was added and incubated for further 2 hours. Finally medium was decanted and the produced insoluble formazan was dissolved in 1 ml DMSO and read against blank (DMSO) at OD540 using a Dynatech Microwell plate reader (Dynatech Labs Inc., Chantilly, VA, USA) for cytotoxicity measurement as before [13–18]. Cell viability was estimated using the following formula: (sample values of OD_540_-blank value)/(NC value of OD_540_-blank value)×100%. In some experiments, cells were pretreated with aspirin, NAC, catalase, SOD, ZnPP, or U0126 for 30 min and then CQ (final 2 mM) was added and co-incubated for 24 hours. Culture medium was collected for ELISA. Cytotoxicity was determined by MTT assay as above.

### Flow Cytometric Analysis of Cell Cycle Distribution and Apoptosis

In short, 2.5 x 10^5^ human dental pulp cells were incubated in each well of a 6-well plate in 2 ml of DMEM with 10% FBS. 24 hours later, the medium was replaced by a fresh one containing 0.4% v/v DMSO (NC) or different concentrations of CQ (0.1, 0.25, 0.5, 1 and 2 mM) and further cultured for 24 hours.

### Cell Cycle Analysis: PI Staining Flow Cytometric Analysis

After exposure of pulp cells to CQ, both the attached and the floating cells were collected. They were resuspended and fixed in 70% ethanol at -20°C for 30 minutes. After centrifugation and washed with PBS, 350 μl of propidium iodide (PI, 40 μg/ml) was added for cell staining and then 2 μl of RNase A was added. After staining, the PI-elicited fluorescence of individual cells was determined by a FACS Calibur Flow Cytometer (Becton Dickinson, Worldwide Inc., San-Jose, CA, USA) [15,19,20]. Totally the PI fluorescence of 10000 cells was counted for each sample. The percentage of cells residing in sub-G_0_/G_1_ phase was measured using the CELL Quest program (Becton Dickinson, CA, USA), while the distribution of cells in G_0_/G_1_, S, and G_2_/M phase were calculated with the ModFit LT program version 2.0 (Verity Software House, Inc., USA).

### Cell Death Pattern Analysis by PI/Annexin V Flow Cytometric Analysis

Both the attached and floating cells were collected together. They were resuspended in 300 μl of incubation buffer and stained with 8 μl of PI (50 μg/ml) and 4 μl of Annexin V for about 30 minutes [15]. Later, the PI- and Annexin V-elicited fluorescence of individual cell was measured using a FACS Calibur Flow Cytometer (Becton Dickinson, Worldwide Inc., San-Jose, CA, USA). A total of 10000 cells were analyzed for each sample. The percentages of stained cells distributed in the lower left [LL: PI(-), Annexin V(-)], lower right [LR: PI(-), Annexin V(+)], upper left [UL: PI(+), Annexin V(-)], and upper right [UR: PI(+), Annexin V(+)] quadrants were determined using the CELL Quest program (Becton Dickinson, CA, USA).

### Cellular ROS Production: Flow Cytometric Analysis of Cellular DCF Fluorescence and the Measurement of Medium 8-Isoprostane Level

Cells were exposed to different concentrations of CQ for 2.5 hours and then stained with DCFH-DA at the concentration of 10 μM for further 30 minutes (total 3 hours). Then, both floating and attached cells were collected together. The cells were then re-suspended in 200 μl of phosphate buffered saline (PBS) and the DCF-elicited fluorescence of individual cells was measured by using a FACS Calibur Flow Cytometer (Becton Dickinson, Worldwide Inc., San-Jose, CA, USA) [13,15,19]. A total of 10000 cells were analyzed for each sample. The mean of DCF fluorescence was determined by using the CELL Quest program (Becton Dickinson, CA, USA). The level of 8-isoprostane in culture medium was measured with ELISA kits.

### Cellular PGE_2_ and 8-Isoprostane Production

For measurement of PGE_2_ production, pulp cells were exposed to CQ for 24 hours. Culture medium was collected for measuring the 8-isoprostane and PGE_2_ production by ELISA. In some experiments, cells were pretreated with ZnPP, and U0126 for 30 min and then CQ (final 2 mM) was added and co-incubated for 24 hours. Culture medium was collected for ELISA analysis of PGE_2_ production [13,21].

### Reverse Transcription-Polymerase Chain Reaction (RT-PCR) for Analysis of mRNA Expression

1.5×10^6^ dental pulp cells were cultured in 10-cm dishes containing 10 ml of DMEM with 10% FBS. Twenty-four hours later, the culture medium was changed to a fresh one containing 0.4% v/v DMSO (NC) or various concentrations of CQ (0.1, 0.25, 0.5, 1 and 2 mM), and incubated for another 24 hours. Then, Total cellular RNA of dental pulp cells was isolated by using the RNA isolation kits. RT-PCR was used to evaluate the mRNA expression of human dental pulp cells.

Briefly denatured RNA (5 μg) was reverse transcribed at 42°C for 90 minutes in a thermal cycler. The reaction mixture (45 μl) contained 4 μl of random primer (500 μg/ml), 8 μl of dNTP (2.5 mM), 4.5 μl of 10x RT buffer, 1 μl of RNase inhibitor (40 U/μl), 0.5 μl of RT (21 U/μl), and double distilled water. The generated cDNA product was used for further PCR amplification in a reaction mixture comprising 5 μl of 10x Super TAQ buffer, 4 μl of 2.5 mM dNTP, 1 μl of each specific primer, 0.2 μl of Super TAQ enzyme (2 U/μl), and double distilled water. The specific primers were: cdc2: GGGGATTCAGAAATT GATCA and TGTCAGAAAGCTACATCTTC (288 bp product), cdc25C: CCTGGTGAGAATTCGAAGACC and GCAGATGAAGTACACAT TGCATC (456 bp), cyclin B1: AAGAGCTTTAAACTTTGGTCTGGG and CTTTGTAAGTCC TTGATTTACCATG (317 bp), p21: GAGCGATGGAACTTCGACTTTGTCACC and CTGAGACTAAGGCAGAAG ATGTAGAGCG (450 bp), HO-1: AAGATTGCCCAGAAAGCCCTGGAC and CCAGAAAGCTGAG (399 bp), COX-2: TTCAAATGAGATTGTGGGAAAATTGCT-30 and 50-AGATCATCTCTGCCTGAGTATCTT (305 bp), and β-actin (BAC, 218 bp): AAGAGAGGCATCCTC ACCCT and TACATGGCTGGGGTGT TGAA as control [13,21, 22]. The amplification procedure for the target genes included 20–35 cycles of PCR, denaturing at 94°C for 1 min, annealing at 55°C for 1 min, and extension at 72°C for 1 min. This was followed by a final incubation at 72°C for 7 min. The PCR products were applied for 1.8% agarose gel electrophoresis, and DNA was stained with ethidium bromide for further photograph and presentation.

### Western Blotting Analysis of Protein Expression

Briefly, 1.5 x 10^6^ human dental pulp cells were seeded into 10-cm culture dishes with 10 ml of DMEM with 10% FBS. After 24 hours, the medium was replaced by a fresh one containing DMSO (vehicle control, NC) or different concentrations of CQ (0.1, 0.25, 0.5, 1, and 2 mM). After 24 hours, cells were washed with PBS and then disrupted in lysis buffer (10 mM Tris-HCl, pH 7; 140 mM sodium chloride; 3 mM magnesium chloride; 0.5% NP-40; 2 mM phenylmethylsulfonyl fluoride; 1% aprotinin; and 5 mM dithiothreitol) [13,23]. Then aliquots (20–50 μg protein) of cell lysates were loaded to 12.5% sodium dodecyl sulfate-polyacrylamide gel electrophoresis (SDS-PAGE) for protein separation and transferred to a polyvinylidene fluoride (PVDF) membrane. The membrane was blotted first with anti-human HO-1, cdc2, cdc25c, cyclin B1, COX-2, p-ERK1/ERK2 and Glyceraldehyde 3-phosphate dehydrogenase (GAPDH) primary antibodies for 2-hr. This was followed by incubation with horseradish peroxidase-link secondary antibodies (Jackson ImmunoResearch Laboratories, West Grove, PA, USA) for 1 hr. After washing the membrane with buffer, ECL reagents (Amersham) were added and the chemiluminescence was detected by exposure of membranes to Fuji films for 30 sec to 10 min. The intensity of GAPDH was used as control.

### Immunofluorescent Microscope Observation of p-ATM, p-Chk2, p53 and Growth Arrest and DNA Damage-45α (GADD-45α) Protein Expression

In brief, 4 × 10^4^ human dental pulp cells were seeded on the sterile coverslips in a 24-well plate in DMEM and 10% FBS. After 24 hours, they were exposed to different concentrations of CQ (0.25, 0.5, 1, 2, 3 mM) for further 24 hours. Medium was decanted, and cells were washed with PBS and fixed in 4% paraformaldehyde for 20 minutes. Cells were washed with PBS, permeabilized with 2% Triton X-100, incubated in 0.3% v/v H_2_O_2_ for 20 minutes. After washed with PBS, cells were blocked in 5% bovine serum albumin (BSA) for 1 hour and then incubated in primary antibodies (p-ATM, p-Chk2, p-p53 and GADD) (1:1000, v/v) at room temperature overnight. After PBS wash, cells were incubated in corresponding secondary antibody (FITC- or TRITC-conjugated) in the dark for 1 hour and counterstained with DAPI (1:1000) for 30 minutes. Finally the samples were mounted and observed/photographed under Olympus IX71 inverted microscope and DP Controller/Manager software (Olympus Corporation).

### Statistical Analysis

All experiments were performed separately for at least 3 times. The means and standard errors (SE) of each experiment were calculated. The differences between experimental and control groups were analyzed by One-way ANOVA and post hoc Tukey test using the SPSS 10.0 software for windows. A p-value < 0.05 was considered to constitute a significant difference between the groups.

## Results

### Cytotoxicity of CQ on Human Dental Pulp Cells

**Fig 1:**
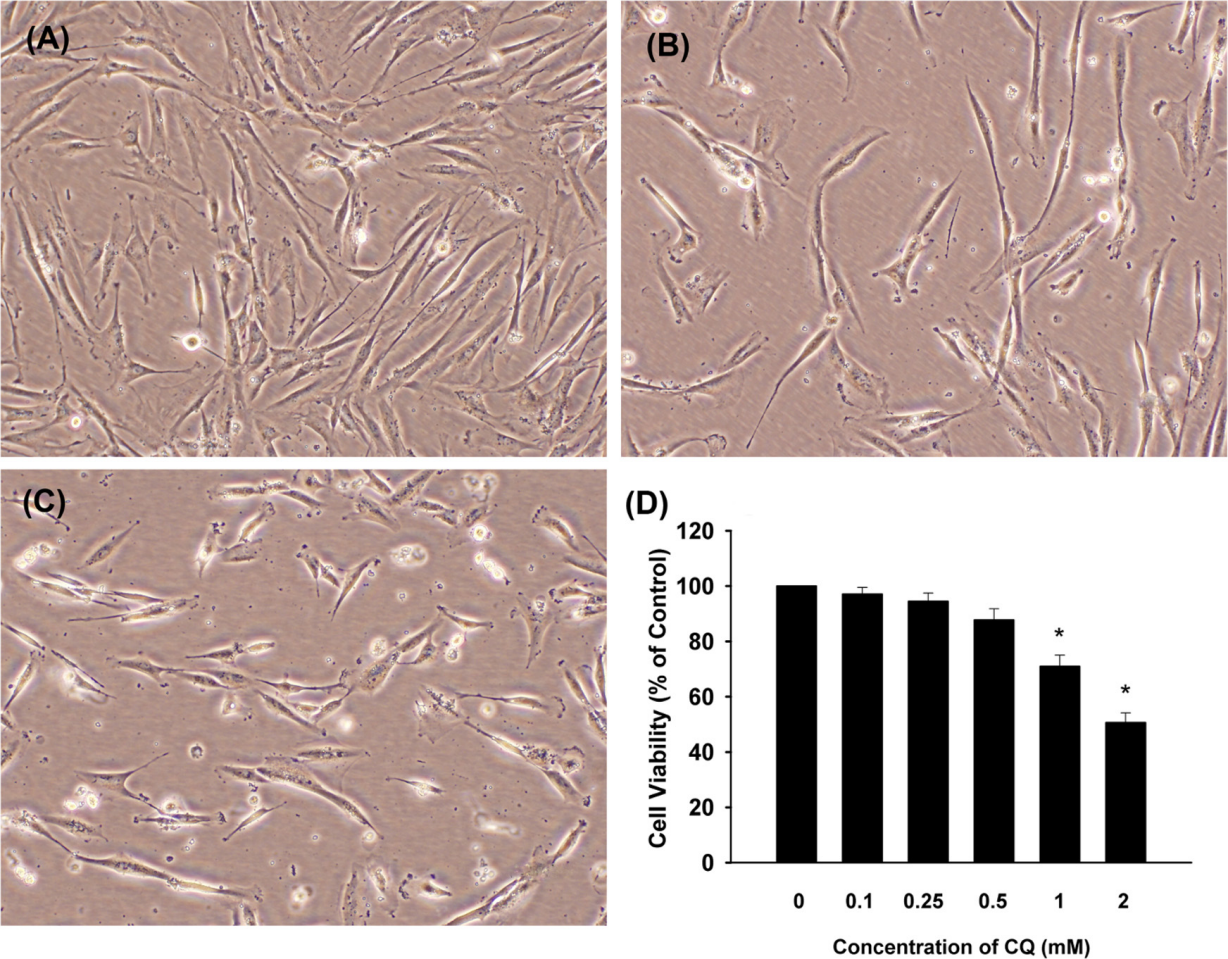
Morphology of human dental pulp cells after exposure to CQ for 24 hours. **(A)** Control (solvent), **(B)** 1 mM CQ, **(C)** 2 mM CQ, (100x, original magnification), **(D)** Quantitative cytotoxicity of CQ on dental pulp cells as analyzed by MTT assay. Results were expressed as % of control (as 100%). *denotes statistically significant difference (p<0.05) when compared with solvent control group.

Human dental pulp cells are spindle-shaped in appearance with extended cellular process when observed under a phase-contrast microscope **(Fig 1A)**. After incubated with lower concentrations of CQ (0.1, 0.25, and 0.5 mM) for 24 hours, the morphology of cells was not altered markedly but the cell density seemed to be a little less than that of control group **(data not shown)**. However, following exposure to higher concentrations of 1 mM and 2 mM CQ for 24 hours, the cells became much sparser in arrangement, smaller, retracted, and even rounded, especially in the CQ 2 mM group **(Fig 1B and 1C)**. Cell proliferation was expressed as the percentage of cells proliferating in the presence of CQ relative to the cells proliferating in the absence of CQ (NC). As shown in **Fig 1D**, CQ inhibited the growth of human dental pulp cells in a dose-dependent manner. Cell viability was significantly reduced to about 70% and 50% of control under the treatment of 1 mM and 2 mM CQ (p<0.05) for 24 hours.

### Effects of CQ on Cell Cycle Progression of Pulp Cells

**Fig 2:**
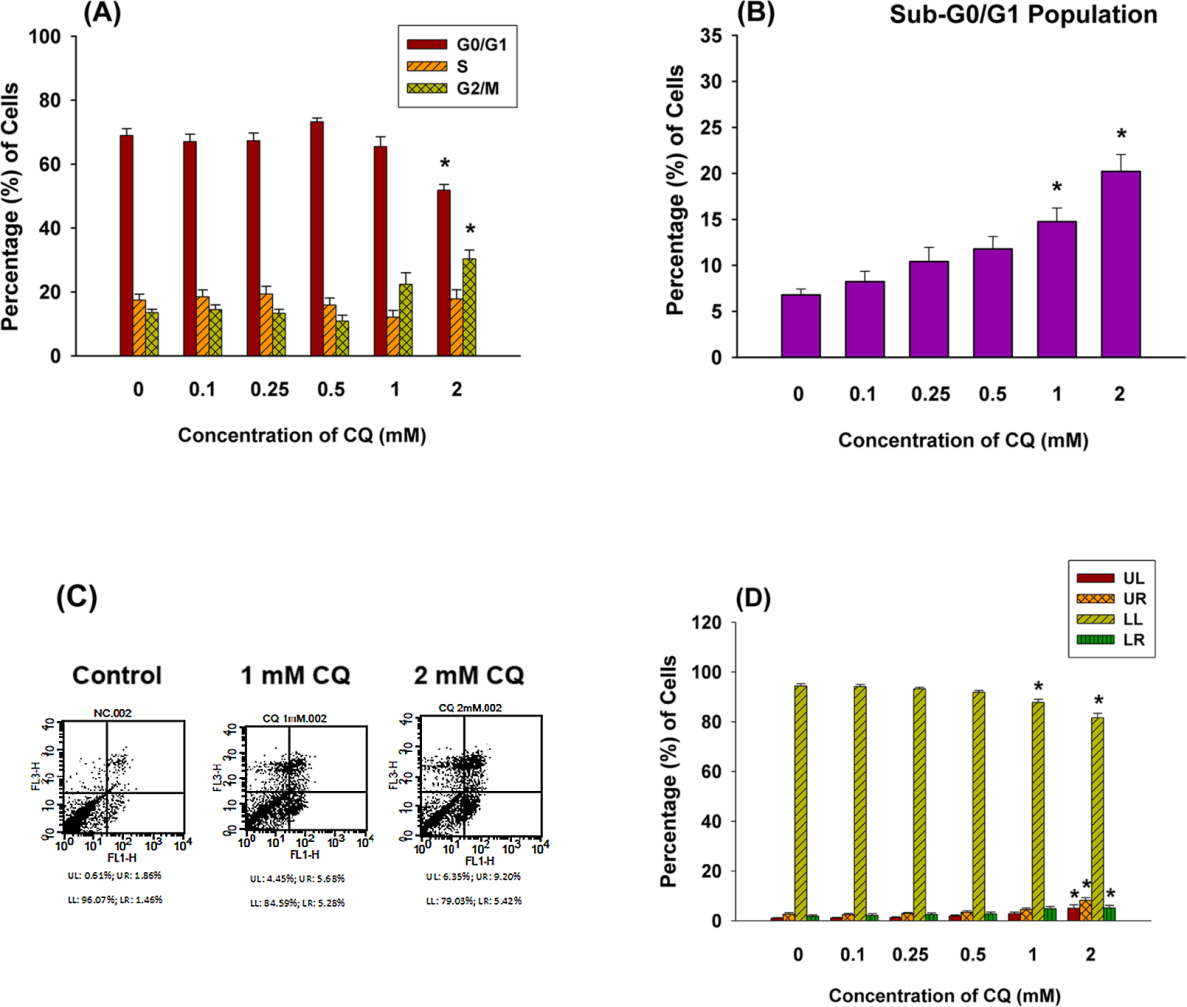
Effect of 24-hours exposure to CQ on the cell cycle progression and apoptosis of pulp cells. **(A)** Quantitative histogram representing the percentage of cells residing in each cell cycle phase (n = 9, Mean±SE, *denotes the presence statistically significant difference when compared with control (p<0.05), **(B)** Quantitative histogram representing the percentage of cells residing in sub-G0/G_1_ phase was shown (n = 9, Mean±SE, * denotes a p-value<0.05) **(C)** Inducing the apoptosis of pulp cells by CQ as analyzed by PI/Annexin V dual staining flow cytometry. One representative PI/Annexin V flow cytometry profiles of pulp cells. Human dental pulp cells were exposed to CQ for 24 hours. In each plot, the lower left quadrant represents viable cells [LL: PI(-), Annexin V(-)], the lower right quadrant represents apoptotic cells [LR: PI(-), Annexin V(+)], the upper left quadrant represents necrotic cells [UL: PI(+), Annexin V(-)], and the upper right quadrant represents primary apoptotic, secondary necrotic cells [UR: PI(+), Annexin V(+)]. **(D)** Quantitative histogram of PI/Annexin V assay (n = 5, Mean±SE, *denotes a statistically significant difference when compared with solvent-treated control (p<0.05).

Human dental pulp cells treated with CQ demonstrated growth arrest. In untreated cells (NC), there were 69%, 17% and 14% of cells residing in G_0_/G_1_, S and G_2_/M phase of cell cycle respectively, with no obvious changes after incubation in 0.1–0.5 mM CQ. Exposure of pulp cells to 1 mM CQ led to a moderate increasing percentage of cells residing in G_2_/M phase (22%, p = 0.067). However, following the exposure to 2 mM CQ for 24 hours, significant G2/M cell cycle arrest was noted as revealed by increasing percentage of cells to 30% (p<0.05) **(Fig 2A).** Besides, evident increase in sub-G_0_/G_1_ peak was noted in 1 mM and 2 mM (p<0.05) CQ groups, indicating the possible induction of cellular apoptosis **(Fig 2B)**. PI-annexin V dual staining flow cytometric analysis further found that there were 94.4%, 1.9%, 1.0%, and 2.7% of cells residing in LL, LR, UL, and UR quadrant respectively in control cells (NC). No marked changes after incubation in 0.1, 0.25, and 0.5 mM CQ for 24 hours. Exposure of pulp cells to 1 mM CQ led to an obvious increased percentage of cells in LR quadrant (pro-apoptotic, 4.8%, p = 0.084). A significant increase of cells in LR (5.2%, p<0.05), UL (necrosis, 4.98%, p<0.05) and UR (apoptotic, 8.21%, p<0.05) quadrant was noted after the exposure of pulp cells to 2 mM CQ **(Fig 2C and 2D).**

### mRNA and Protein Expression of Cell Cycle-Related Genes

**Fig 3:**
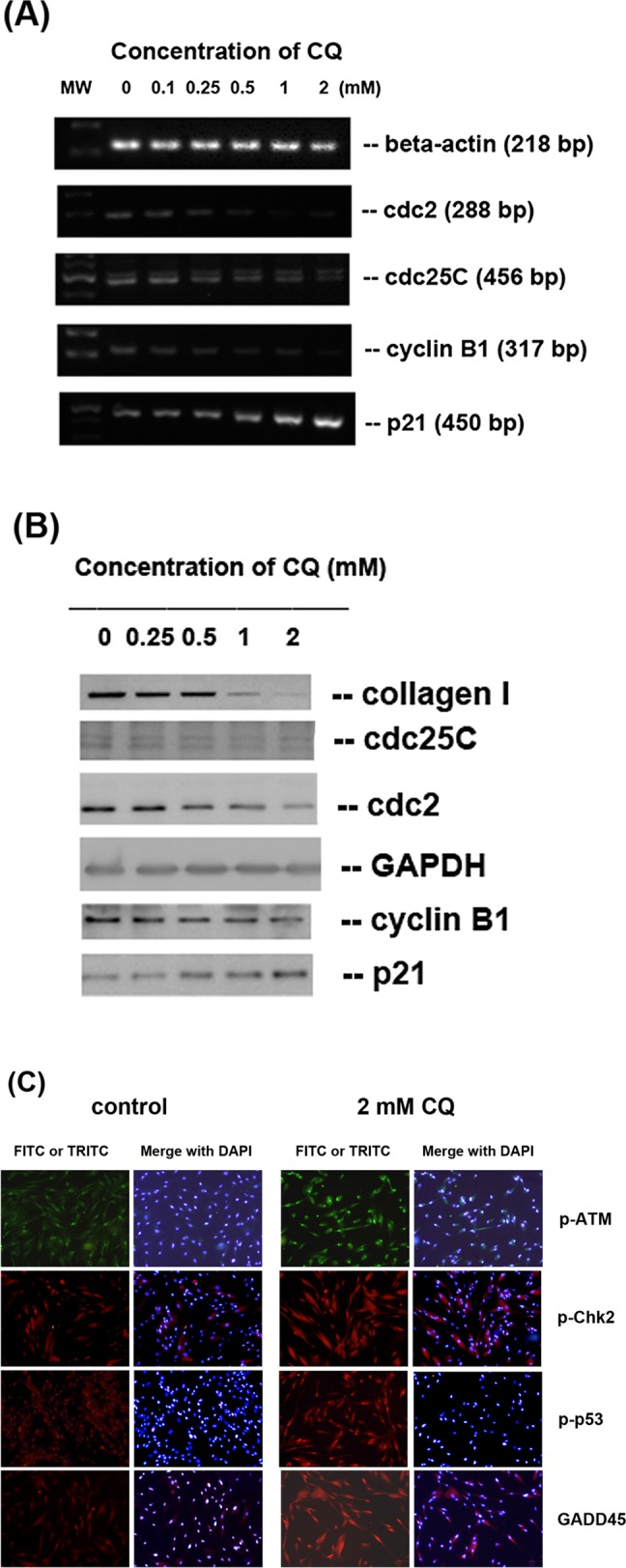
Effects of CQ on cell cycle-related genes and protein expression of human dental pulp cells. Human dental pulp cells were treated with CQ for 24 hours. **(A)** mRNA expression by RT-PCR analysis (cdc2, cdc25C, cyclin B1, p21 and beta-actin as control), **(B)** Western blotting analysis of protein expression (cdc2, cdc25C, cyclin B1, p21, collagen and GAPDH as control). **(C)** Immunofluorescent staining of p-ATM (FITC, green), p-Chk2, p-p53 and GADD45 (TRITC, red) protein expression of pulp cells with/without exposure to 2 mM CQ for 24 hours. Cell nuclei were stained with DAPI (as blue fluorescence). One representative picture of RT-PCR, western blotting and IF was shown.

Since exposure to CQ led to cell cycle arrest, further evaluation was done for the expression of cell cycle G2/M phase-related genes, including cdc2, cdc25C, cyclin B1 and p21. Results indicated that CQ (> 0.25 mM) inhibited cdc2, cdc25C and cyclin B1 expression of pulp cells. However, CQ induced p21 mRNA expression of pulp cells **(Fig 3A)**. We also evaluated the expression of related proteins by western blot analysis. We found that CQ inhibited cdc2, cdc25C and cyclin B1 protein expression of pulp cells, whereas CQ stimulated p21 protein expression **(Fig 3B)**. Moreover, CQ also inhibited the expression of type I collagen, the important extracellular protein of pulp **(Fig 3B)**. Immunofluorescent staining further showed increasing ATM phosphorylation (p-ATM) in the nucleus of pulp cells after exposure to CQ **(Fig 3C)**. The ATM-phosphorylated p-Chk2 (red TRITC fluorescence) also increased in both nucleus and cytosol after exposure to 2 mM CQ **(Fig 3C)**. The expression of p-p53 (red TRITC fluorescence) was slightly and gradually increased in the nucleus of pulp cells after incubation in 2 mM CQ **(Fig 3C)**. A p53-regulated protein, GADD45α (red TRITC fluorescence), was also boosted at 2 mM CQ. The expression of GADD45α was extensive in the cytoplasm and nucleus **(Fig 3C)**.

### Effects of CQ on Oxidative Stress of Human Dental Pulp Cells

**Fig 4:**
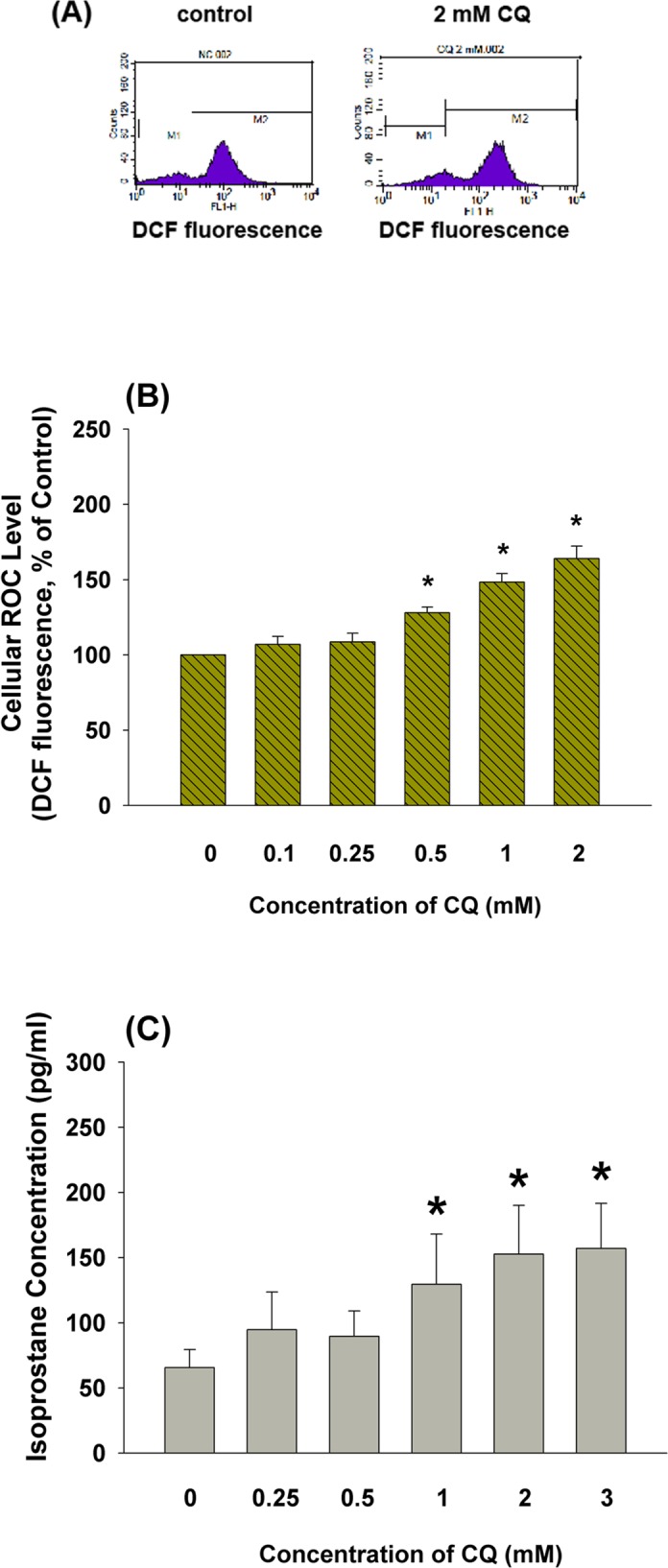
Effect of CQ on cellular oxidative stress of dental pulp cells. **(A)** One representative DCF flow cytometry profiles of pulp cells was shown. Human dental pulp cells were exposed to CQ for 3 hours. In each plot, there were 2 populations of cells, M1 and M2, with different intracellular DCF content. The mean fluorescence of cells in M2 population represented the DCF fluorescence of that group. **(B)** Quantitative histogram of DCF assay (n = 5, Mean±SE, *denotes a statistically significant difference when compared with solvent-treated control (p<0.05), **(C)** Stimulation of 8-isoprostane production of pulp cells by CQ. *denotes statistically significant difference when compared with control.

The fluorescence of DCF was expressed as the mean fluorescence of cells exposed to CQ relative to those in control group (control near 100). As shown in **Fig 4A**, exposure to 2 mM CQ elevated the intracellular ROS as revealed by the increase in DCF fluorescence. Quantitatively, exposure to higher concentrations of CQ for 3 hours led to a significant concentration-dependent increase of intracellular ROS. The mean DCF fluorescence increased markedly to about 128.02%, 148.38% and 164.12%, respectively, by the treatment of 0.5, 1 and 2 mM CQ (p<0.05) for 3 hours **(Fig 4B)**. Since 8-isoprostane has been shown to be an oxidative stress marker, we further studied and found the stimulation of 8-isoprostane production by CQ in dental pulp cells at concentrations of 1–3 mM **(Fig 4C)**.

### Effects of CQ on COX-2 Expression and PGE_2_ Production of Pulp Cells

**Fig 5:**
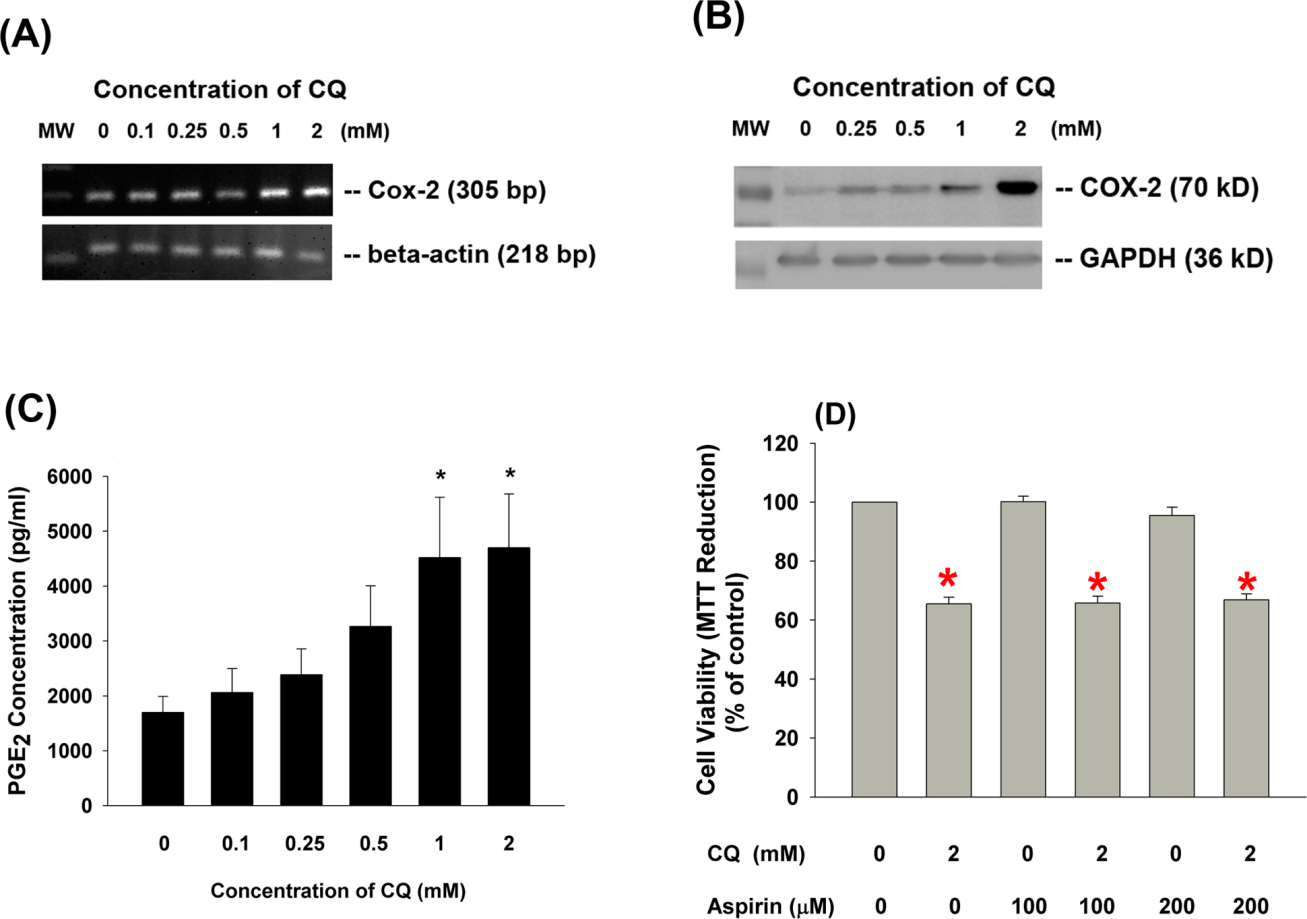
Effects of CQ on COX-2 expression and PGE_2_ production of human dental pulp cells. Human dental pulp cells were treated with CQ for 24 hours. **(A)** COX-2 mRNA expression of pulp cells, **(B)** COX-2 protein expression of pulp cells, **(C)** PGE_2_ production of pulp cells. **(D)** Effect of aspirin on CQ-induced cytotoxicity of pulp cells. Results were expressed as Mean±SE. *denotes statistically significant difference when compared with solvent control.

Since the exposure of dental pulp to composite resin or DBA may potentially induce pulpal inflammation, it is interesting to know whether CQ may induce COX-2 expression and PGE_2_ production. Results showed that CQ stimulated COX-2 mRNA expression at concentrations higher than 1 mM **(Fig 5A)**. Similarly, CQ also induced COX-2 protein expression at concentrations of 1–2 mM **(Fig 5B)**. Accordingly, CQ also stimulated PGE_2_ production of pulp cells at concentrations of 1 and 2 mM **(Fig 5C)** as well as PGF_2α_ production **(data not shown)**. However, inhibition of COX by aspirin was not able to prevent the CQ-induced cytotoxicity to pulp cells **(Fig 5D)**.

### Effects of NAC, Catalase and SOD on CQ-Induced Cytotoxicity of Pulp Cells

**Fig 6:**
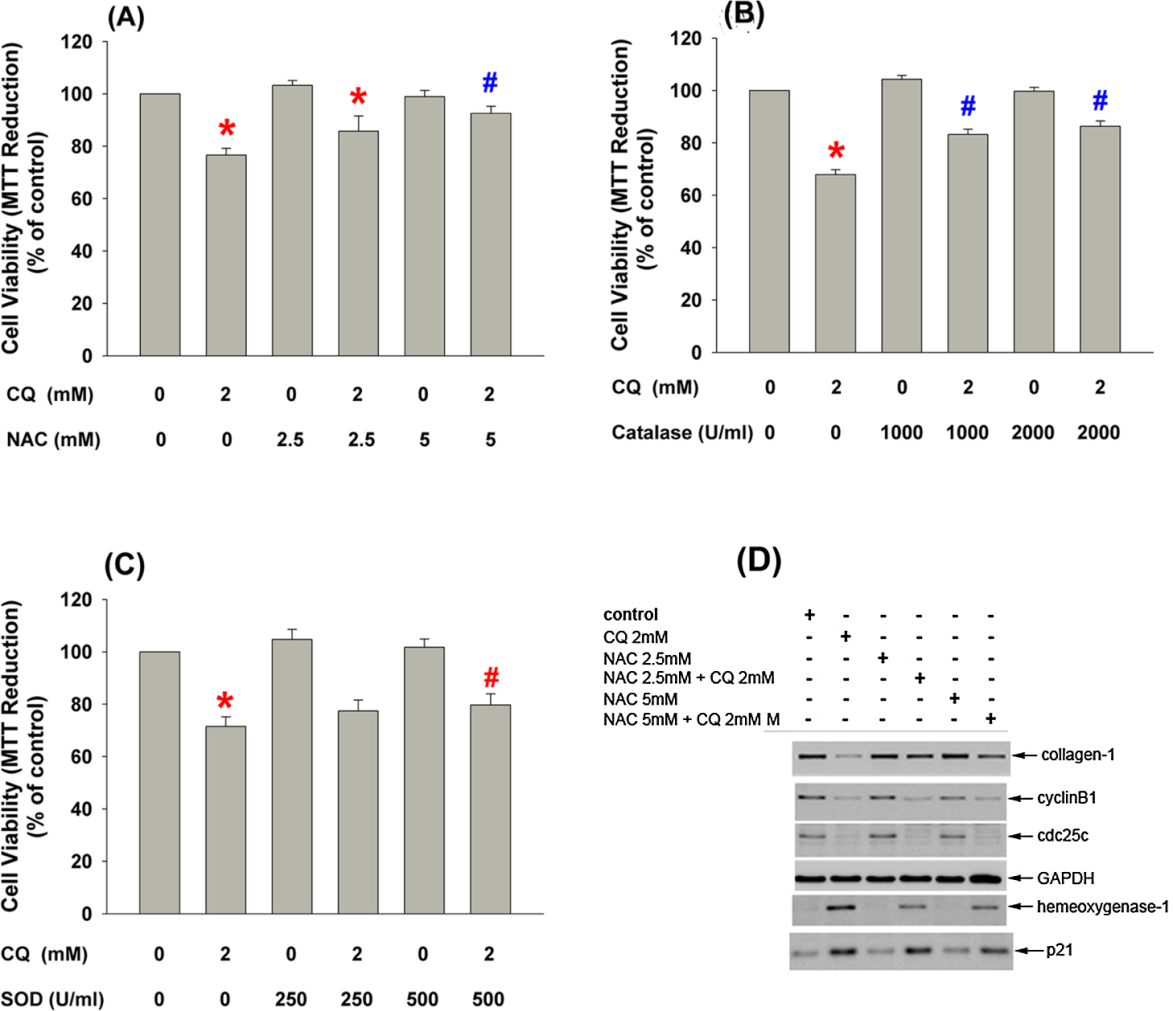
Effect of NAC, catalase and SOD on CQ-induced cytotoxicity of pulp cells (A) Effect of NAC on CQ-induced cytotoxicity of pulp cells, (B) Effect of catalase on CQ-induced cytotoxicity of pulp cells, (C) Effect of SOD on CQ-induced cytotoxicity of pulp cells. Results were expressed as cell viability (Mean±SE, % of control), *denotes a statistically significant difference when compared with solvent-treated control. #denotes a statistically significant difference when compared with CQ solely group (p<0.05). (D) Effect of NAC on CQ-induced changes of p21, collagen I, cdc2, cdc25C and cyclin B1 protein expression. GAPDH expression was used as control. One representative western blotting result was shown.

Cell viability was expressed as the percentage of control. After pre-treatment with 2.5 and 5 mM NAC for 30 minutes and then co-incubation with 2 mM CQ for 24 hours, the reduction of cell number caused by 2 mM CQ was significantly attenuated (p<0.05) **(Fig 6A)**. Besides, the decrease in viable cell number by CQ was also prevented by the pre-treatment and co-incubation with catalase **(Fig 6B)**. SOD (500 U/ml) also decreased the CQ-induced cytotoxicity to pulp cells **(Fig 6C)**. Moreover, NAC attenuated the CQ-induced p21 and HO-1 protein expression, reversed the CQ-induced decline of collagen I expression, but showed little effect on CQ-induced decrease in cyclin B1, cdc2 and cdc25C expression of pulp cells **(Fig 6D)**.

### Stimulation of HO-1 by CQ in Dental Pulp Cells and Its Role in Toxic Effect

**Fig 7:**
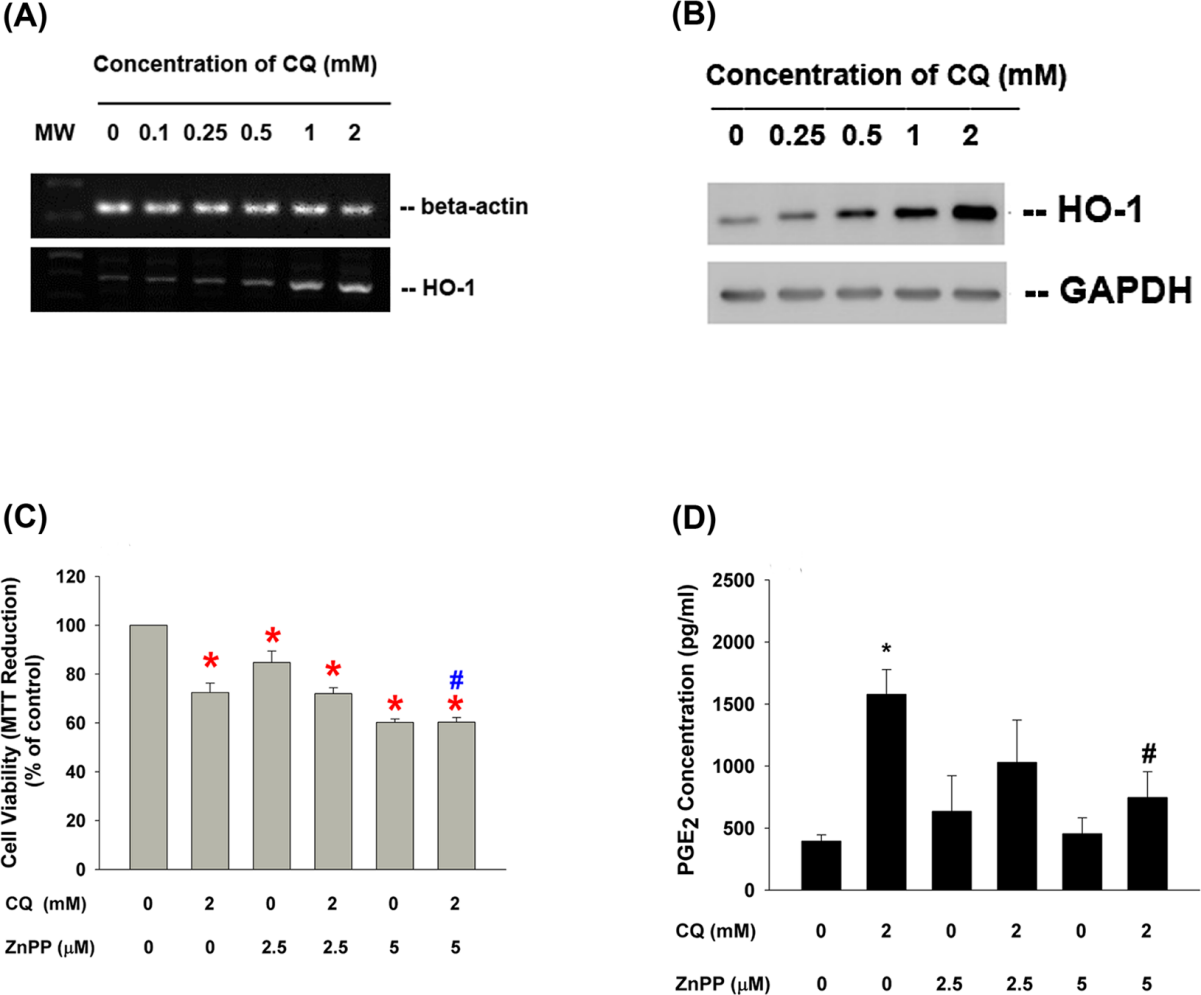
CQ induced HO-1 expression and its role in cytotoxicity, isoprostane, and PGE2 production of pulp cells. **(A)** Stimulation of HO-1 mRNA expression by CQ in dental pulp cells. **(B)** Stimulation of HO-1 protein expression by CQ in dental pulp cells. **(C)** Effect of ZnPP on CQ-induced cytotoxicity, **(D)** Effect of ZnPP on CQ-induced PGE_2_ production of pulp cells. *denotes the presence of statistically significant difference when compared with control. #denotes a statistically significant difference when compared with CQ solely group (p<0.05).

The expression of HO-1 was weak in control pulp cells. CQ (> 0.5 mM) markedly stimulated HO-1 mRNA expression of pulp cells as analyzed by RT-PCR **(Fig 7A)**. Similarly CQ also induced HO-1 protein expression at concentrations higher than 0.5 mM, and even more obvious at 1–2 mM **(Fig 7B)**. Moreover, the reduction of cell viability by 2 mM CQ was markedly enhanced after the pre-treatment/co-incubation with 5 μM ZnPP (a HO-1 inhibitor) **(Fig 7C)**. Accordingly, the CQ-induced PGE_2_ production of pulp cells was also inhibited by ZnPP **(Fig 7D)**, possibly due to cytotoxicity.

### Role of MEK/ERK Signaling in Regulation of CQ-Induced Toxicity

**Fig 8:**
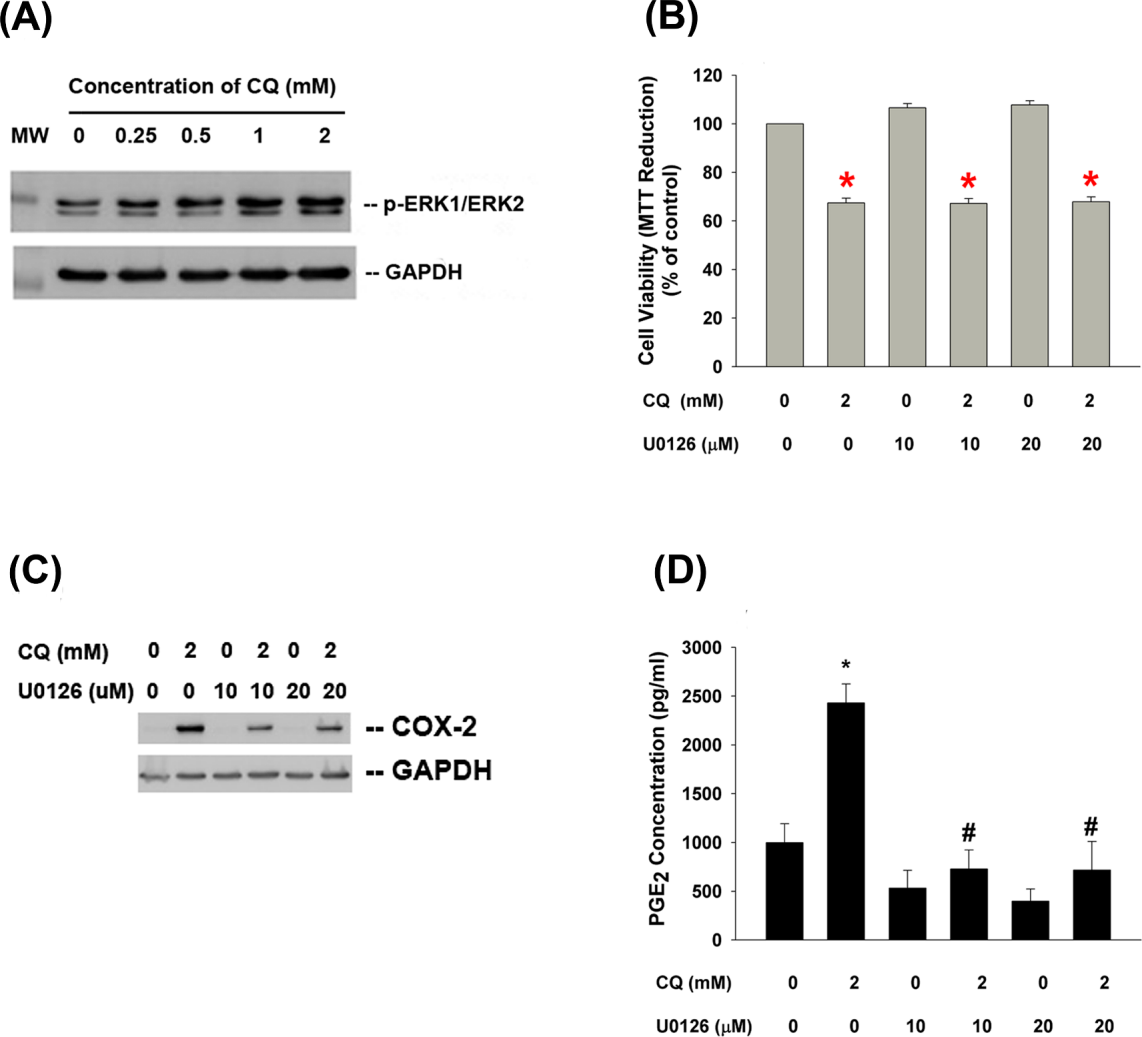
Role of MEK/ERK signaling on CQ-induced cytotoxicity, COX-2 expression and PGE_2_ production of pulp cells. **(A)** Stimulation of ERK1/ERK2 phosphorylation by CQ, **(B)** Effect of U0126 on CQ-induced cytotoxicity of pulp cells, **(C)** Effect of U0126 on CQ-induced COX-2 protein expression of pulp cells. One representative western blotting result was shown. **(D)** Effect of U0126 on CQ-induced PGE_2_ production of pulp cells. Results were expressed as Mean±SE, *denotes a statistically significant difference when compared with solvent-treated control. #denotes a statistically significant difference when compared with CQ solely group (p<0.05).

In order to know the signal transduction pathways responsible for the CQ-induced cellular alteration, we first studied the MEK/ERK signaling, and found that CQ stimulated ERK1/ERK2 phosphorylation of pulp cells at concentrations higher than 0.25 mM **(Fig 8A)**. U0126 (a MEK/ERK signaling inhibitor) was not able to prevent the CQ-induced cytotoxicity **(Fig 8B)**. Intriguingly, U0126 could attenuate the CQ-induced COX-2 protein expression and PGE_2_ production of pulp cells **(Fig 8C and 8D)**.

## Discussion

It has been found that photoinitiators and monomers may be released from dentin bonding agents and various resinous materials even after light curing. They may potentially affect the health of localized oral mucosa when released into saliva or the dental pulp tissues when the caries cavity is deep with thin remaining dentin thickness [24,25]. Destructive effects such as mineralization impairment, inflammation, abscess formation, and necrosis of the dental pulp are found when deep caries cavities are filled by DBA and resin composites [26–28]. The effect of photoinitiators and resin monomer on dental pulp cells may convey crucial clinical implications, but the underlying mechanisms await further investigations. In the present study we found one popularly-used photoinitiator, CQ, inducing marked cytotoxicity to human dental pulp cells at concentrations higher than 1 mM, and with morphological alterations, which are known to be crucial for cell proliferation, movement, as well as tissue morphogenesis [23]. These findings are in concordance with the morphological aberrations induced by hydroxyethylmethacrylate (HEMA), a common monomer in resin composites and dentin bonding agents (DBAs). Chang *et al*. presented apparent retraction and loss of the extended cellular process after treated with 10 mM HEMA for 24 hours [19]. Accordingly the proliferation of human dental pulp cells was obviously suppressed to about 70% and 50% of control after exposure to CQ (1–2 mM) for 24 hours, respectively. This observation is similar to the data reported on gingival fibroblasts [1,8]. Masuki *et al*. documented that the effective inhibitory concentration of CQ on human gingival fibroblasts was 1 mM [1], while Volk *et al*. reported that the ED_50_ (effective dose, 50%) value of CQ to human gingival fibroblasts was about 2.7±0.8 mM [8].

The cytotoxicity by CQ can be partly explained by its induction of cell cycle arrest and apoptosis. Cell proliferation is tightly regulated by cell cycle progression and impairment of cell cycle control may result in growth arrest, cytotoxicity and even apoptosis [12]. Analysis of cell cycle alterations was therefore conducted to explore the cytotoxic mechanism of CQ. We interestingly found that CQ induced cell cycle arrest of human dental pulp cells. A G0/G1 cell cycle arrest was noted in cells treated with 0.5 mM CQ. In addition, higher concentrations of CQ (1 an 2 mM) induced G2/M cell cycle arrest. Sub-G0/G1 population elevated in 1 and 2 mM CQ-treated groups, suggesting the presence of apoptotic cells. The induction of pulp cell apoptosis by CQ was further confirmed by the PI-Annexin V staining flow cytometry. Masuki *et al*. showed that 1 mM and 5 mM CQ induced a significant and sustained accumulation of human gingival fibroblasts in G_0_/G_1_ phase of the cell cycle after incubation for 24 hours [1]. The possible reasons for the difference may be due to different detoxifying enzymes in human dental pulp cells and human gingival fibroblasts. However, further studies are needed to make clear these points.

Little is known about the mechanisms responsible for CQ cytotoxicity. Cell cycle control and apoptosis are affected by the expression of various cyclins and cyclin-dependent kinases (Cdk) in specific cell cycle phases. A number of proteins are involved in cell cycle checkpoints to respond to DNA damage, recruit repair machinery, delay or arrest cell cycle progression, and even induce apoptosis [10]. For example, G_2_/M transition is regulated by the mitosis promoting factor composed of cdc2 (CDK1) and cyclin B1. In this study, a marked dose-dependent inhibition of the expression of both cdc2 and cyclin B1 was observed after 24 hours exposure to CQ. In addition, CQ also inhibited the expression of type I collagen, a major extracellular protein of dental pulp, suggesting the effect of CQ on matrix turnover and pulpal repair. The inhibitory effect of CQ on cdc2 and cyclin B might therefore down-regulate the association of the mitosis promoting factor, and prevent the cell cycle progression, causing the human dental pulp cells to arrest in G_2_/M phase. The mitosis promoting factor cdc2/cyclin B1 complex is kept inactive during G_2_ phase by kinases Wee1 and Myt1 through phosphorylation on tyrosine 15 and threonine 14 of cdc2. At the onset of mitosis, the phosphatase cdc25C can activate cdc2/cyclin B complex via dephosphorylation of these residues on cdc2 [29]. Besides, it is known that cdc25C is the target of the acute and transient cell cycle delay for G_2_/M phase.

In this study, CQ showed an inhibitory effect to cdc25C expression and stimulation of p21 that might inactivate cdc2/cyclin B1 complex. CQ might therefore induce G2/M arrest and apoptosis of pulp cells possibly via stimulation of p21 and inhibition of cdc2, cyclin B1 as well as cdc25C. In order to know more about the mechanisms of cell cycle arrest and apoptosis induced by CQ, we analyzed the changes in expression of upstream signaling molecules such as GADD, p53, Chk2 and ATM in dental pulp cells. As for the delayed and sustained cell cycle arrest for G_2_/M phase, p53 can be phosphorylated and activated by Chk2 or directly by ATM. The transcriptional targets of p53 include p21, GADD45 as well as 14-3-3σ, and they inactivate the cdc2/cyclin B complex through different ways [30]. The sustained pathway for prevention of cell mitosis is p53-dependent, in which the transcription of p21, GADD45 and 14-3-3σ proteins is activated by p53, thereby inactivating or sequestering cyclin B/cdc2 complex [29]. In this study, GADD45α protein expression was also stimulated by CQ. GADD45 bond both Cdks and proliferating cell nuclear antigen (PCNA), a protein involved in DNA replication and repair. GADD45 may inhibit cell cycle progression and promote DNA excision repair. This was possibly due to activation of upstream p53 molecule that may occur in response to DNA damage, hypoxia or other genotoxic stress. We further found the activation of ATM, Chk2 and p53 of pulp cells by CQ, indicating the triggering of DNA double strand breaks or other damages to pulp cells by CQ. Similarly Eckhardt et al. [31] have reported that TEGDMA may stimulate ROS production, induce DNA damage, and activate ATM, p38 and ERK in THP-1 mononuclear cells to control cell survival and apoptosis. These results suggest the possible cytotoxic and genotoxic effect to dental pulp during clinical composite resin restoration procedures.

Since pulpal inflammation was often noticed when exposed pulp or deep caries cavity is restored with DBA and composite resins, we further studied and found that CQ stimulates prostaglandin E_2_ (PGE_2_) and PGF_2α_ production of pulp cells. Prostaglandins (PGs) are important mediators to regulate pulp tissue inflammatory response [32], and prior study has found an increasing level of PGE_2_ and PGF_2α_ in the diseased processes of pulpitis [33]. DBA and resinous root canal sealers are also demonstrated to stimulate COX-2 expression in human osteoblasts [34]. Healthy dental pulp expresses little COX-2, whereas all inflamed dental pulps reveal elevated COX-2 expression [35]. As a major component of DBA and resin composites, CQ (>0.5 mM) is shown to induce COX-2 mRNA and protein expression in human dental pulp cells in this study. To the best of our knowledge, this is the first study to show stimulatory effect of CQ on COX-2 expression and prostanoids production. Both PGE_2_ and PGF_2α_ may involve in the pulpal inflammatory response possibly via activation of prostaglandin EP and FP receptors, as well as stimulation of calcium and MEK/ERK signaling to affect pulpal functions, such as alkaline phosphatase activity and IL-8 production [14,16,21]. Addition of aspirin cannot prevent the CQ-induced cytotoxicity, further suggesting that COX activation is not involved in this process. These results suggest a potential role of CQ in inducing pulpal inflammation after composite resin restoration of dental caries.

Imbalance between ROS and antioxidants may lead to cellular oxidative stress that is crucial for induction of tissue inflammation, degenerative diseases, aging and carcinogenesis [36]. In this study, ROS production increased significantly after the exposure to 0.5–2 mM CQ for 3 hours as shown by elevation of cellular DCF fluorescence and 8-isoprostane production. This finding was generally in accordance to the published data [7,8]. Engelmann *et al*. reported that at concentrations higher than 1 mM, CQ caused a significant concentration-dependent increase of intracellular ROS after 1.5 hours [7]. On the other hand, Volk *et al*. found that at concentrations higher than 0.5 mM, CQ caused a significant concentration-dependent increase of intracellular ROS after 3 hours [8]. The mechanisms and the role of 8-isoprostane production in pulp cells and its role in regulation of pulpal physiology and pathological processes awaited further investigation. Since ROS production had been shown to mediate resin monomers-induced toxicity [25] and anti-oxidants had a potential to decrease their toxic effects [15,25], we further delineated the relationship between ROS formation and cytotoxicity caused by CQ. Interesting, three ROS scavengers—NAC, catalase and SOD, all attenuated the CQ-induced cytotoxicity to dental pulp cells, suggesting the involvement of ROS in CQ cytotoxicity. Unexpectedly, NAC could attenuate the CQ-induced p21 and HO-1 protein expression, preventing the CQ-induced decline of collagen I expression, but showed little effect on CQ-induced decrease in cyclin B1, cdc2 and cdc25C expression of pulp cells. These results suggested that excessive amounts of ROS may lead to cell death via p21 and inhibit collagen formation that can be prevented by NAC/catalase/SOD. A small elevation of cellular ROS was enough to inhibit cyclin B1, cdc2, cdc25C expression, whereas excessive amounts of ROS were needed to stimulate p21, HO-1 and inhibit collagen I that can be more easily prevented by NAC.

HO-1 is known to be an inducible isoform of heme oxygenase in response to oxidative stress and is a protective enzyme against oxidant-induced injury [9]. HO-1 may catalyze the decomposition of heme to generate iron, carbon monoxide and biliverdin, which have the activity to promote cell viability, circulatory integrity and inhibit inflammation [37]. Intriguingly, we found that CQ (> 0.5 mM) stimulated HO-1 mRNA and protein expression, suggesting an adaptive response of dental pulp to CQ. Furthermore, we analyzed the role of HO-1 expression on CQ-induced cytotoxicity by pretreatment and co-incubation with/without ZnPP, a HO-1 inhibitor. We found that the reduction of cell proliferation caused by 2 mM CQ was markedly enhanced by 5 μM ZnPP, indicating the increase of HO-1 expression might play a protective role against CQ-induced cytotoxicity. This may also partly explain the inhibition of CQ-induced PGE_2_ production of pulp cells by ZnPP.

Recently resin monomer—HEMA has been shown to induce ERK activation, but to inhibit protein kinase B (Akt) activity of pulp cells, and both of them may affect the cytotoxicity of HEMA [38]. Suppression of ERK and phosphatidylinositol 3 kinase/Akt signaling pathways also exerts differential effects on the triethylene glycol dimethacrylate (TEGDMA)-induced cytotoxicity to pulp cells [39]. Stimulation of ROS production by HEMA and TEGDMA further activates ERK1/2, JNK and p38 to regulate the cytotoxicity and apoptosis of salivary gland cells [40]. All the above suggest the involvement of mitogen-activated protein kinases (MAPKs) and PI3K/Akt signaling pathways in the resin monomers- and DBAs-induced cell alterations. We further tested whether CQ-induced COX-2 expression and PGE_2_ production was mediated by MEK/ERK1/2 signaling. Intriguingly CQ induced ERK phosphorylation of pulp cells and pretreatment/co-incubation with U0126, a MEK/ERK signaling inhibitor, and markedly suppressed the CQ-induced COX-2 expression and PGE_2_ production. However, U0126 showed little preventive effect on CQ-induced cytotoxicity. This suggested that CQ-induced COX-2 expression and PGE_2_ production of pulp cells were mediated by MEK/ERK signaling. CQ induced ROS generation to mediate cytotoxicity by pathways other than MEK/ERK.

## Conclusions

**Fig 9:**
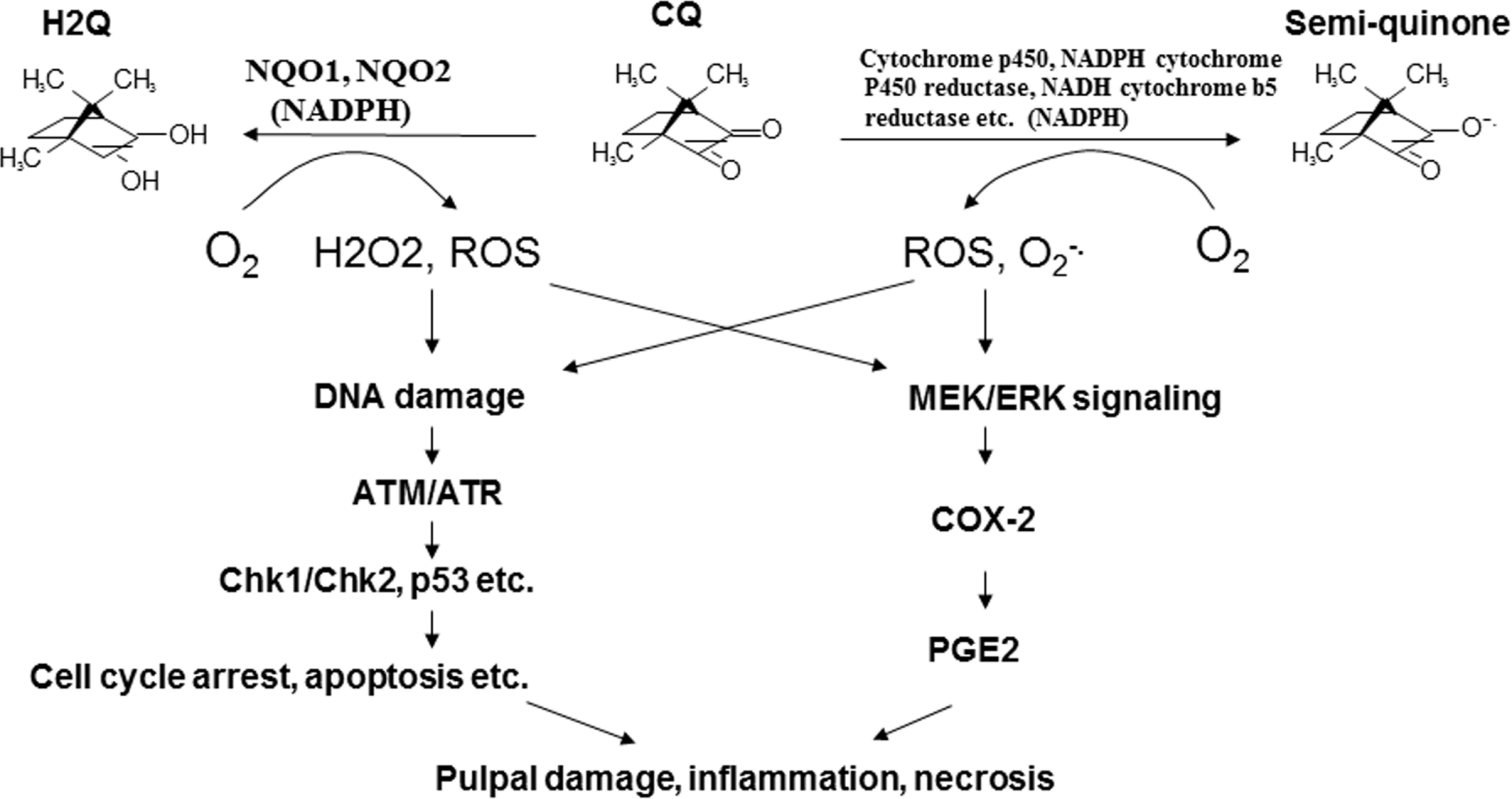
Mechanisms of CQ-induced cytotoxicity and prostaglandins production in dental pulp cells. CQ can be metabolized by one electron reduction enzymes (NQO1, NQO2) or 2 electron reduction enzymes (cytochrome P450, NADPH cytochrome P450 reductase etc.) to generate hydroquinone and semiquinone radicals with concomitantly production of ROS (H_2_O_2_, superoxide etc.). These ROS may involve in cytotoxicity and tissue inflammation via stimulation of DNA damage, ATM/Chk2/p53 and MEK/ERK signaling.

In conclusion, CQ may cause cytotoxicity, cell cycle arrest, apoptosis and PGE_2_ production of pulp cells. These can be due to stimulation of ROS and isoprostane production, activation of ATM/Chk2 signaling, induction of HO-1, COX-2 and p21 expression, and the inhibition of cdc2, cdc25C, cyclin B1 **(Fig 9).** These results are important for our understanding of the mechanism of pulp necrosis and tissue inflammation after clinical operative restoration of dental caries by dentin bonding agent and composite resin.
